# Lymphocytic choriomeningitis arenavirus utilises intercellular connections for cell to cell spread

**DOI:** 10.1038/s41598-024-79397-w

**Published:** 2024-11-22

**Authors:** Owen Byford, Amelia B. Shaw, Hiu Nam Tse, Alex Moon-Walker, Erica Ollmann Saphire, Sean P. J. Whelan, Martin Stacey, Roger Hewson, Juan Fontana, John N. Barr

**Affiliations:** 1 School of Molecular and Cellular Biology, Faculty of Biological Sciences, University of Leeds, Leeds, LS2 9JT UK; 2 Astbury Centre for Structural Molecular Biology, University of Leeds, Leeds, LS2 9JT UK; 3 Center for Infectious Disease and Vaccine Research, La Jolla Institute for Immunology, La Jolla, CA 92037 USA; 4 Department of Molecular Microbiology, Washington University in St. Louis, St. Louis, MO 63110 USA; 5 Program in Virology, Harvard Medical School, Boston, MA 02115 USA; 6 Virology and Pathogenesis Group, National Infection Service, Public Health England, Porton Down, SP4 0JG UK; 7 Present Address: Merck Research Laboratories, Merck & Co, Cambridge, MA 02141 USA

**Keywords:** Arenavirus, LCMV, Tunnelling nanotubes, Intercellular transmission, Arenaviruses, Viral membrane fusion, Viral transmission

## Abstract

The *Arenaviridae* family of segmented RNA viruses contains nearly 70 species with several associated with fatal haemorrhagic fevers, including Lassa, Lujo and Junin viruses. Lymphocytic choriomeningitis arenavirus (LCMV) is associated with fatal neurologic disease in humans and additionally represents a tractable model for studying arenavirus biology. Within cultured cells, a high proportion of LCMV spread is between directly neighbouring cells, suggesting infectivity may pass through intercellular connections, bypassing the canonical extracellular route involving egress from the plasma membrane. Consistent with this, we visualized abundant actin- and tubulin-rich connections conjoining LCMV-infected and uninfected cells within cultures, resembling tunnelling nanotubes (TNTs). Within these TNT-like connections, confocal and STED microscopy identified puncta containing the major structural components of LCMV virions alongside genomic RNA, consistent with intercellular transit of assembled virions or ribonucleoprotein genome segments. Blocking the extracellular route of infection by adding potent LCMV neutralising antibody M28 to supernatants during infection revealed around 50% of LCMV transmission was via intercellular connections. These results show arenaviruses transmission is more complex than previously thought involving both extracellular and intercellular routes.

**Supplementary Information:**

The online version contains supplementary material available at 10.1038/s41598-024-79397-w.

## Introduction

The *Bunyaviricetes* class of segmented RNA viruses comprises over 500 named viruses divided into 15 families, of which the *Arenaviridae* family currently contains 69 species subdivided into five genera: *Antennavirus*, *Hartmanivirus*, *Innmovirus*, *Mammarenavirus* and *Reptarenavirus*^1^. Mammarenaviruses are designated as Old World (OW) or New World (NW) viruses based on geographical location of their isolation and prevalence, which also correlates with distinctive genetic lineage^2^. Several mammarenaviruses cause serious human disease, such as the OW Lassa virus (LASV) and NW Junín virus (JUNV)^1,2^, both notable for their association with fatal haemorrhagic fevers^3^. Currently, no specific antiviral therapeutics or FDA-approved vaccines exist to target any member of the *Arenaviridae* family^4^. Together, these factors have contributed to LASV, JUNV and other arenaviruses being defined as hazard group 4 pathogens, requiring the highest biosafety level (BSL) 4 containment.

The prototypic species within the *Mammarenavirus* genus is the OW lymphocytic choriomeningitis virus (LCMV; and formally, *Mammarenavirus choriomeningitidis*) for which rodents are the main vector, with the common house mouse *Mus musculus* acting as primary host^2^. Rodent-to-human LCMV transmission frequently occurs, yet severe disease only arises in rare cases. This is most often in immunocompromised patients or neonates, where infection can result in aseptic meningitis^5,6^, with LCMV described as an under-recognised agent of neurological disease^6^. Specifically, the LCMV Armstrong strain is a hazard group 2 virus, requiring only BSL-2 containment, thus acting as a relevant research model for the more pathogenic species of the *Mammarenavirus* genus, due to similarities in structure and function.

Within mature virions, the viral-associated RNA (vRNA) is coated by the viral nucleoprotein (NP) and interacts with the viral RNA-dependent RNA polymerase (RdRp, L protein) to form the viral ribonucleoproteins (RNPs). These are surrounded by a lipid bilayer, which is lined by the viral matrix (Z) protein and contains protruding viral spikes. All species within the *Mammarenavirus* genus have genomes consisting of two segments, small (S) and large (L), which together encode four structural proteins. All species of this genus express their genes using an ambi-sense strategy; initially, input viral RNA (vRNA) S and L segments are transcribed by the viral RdRp, following entry and uncoating, to generate mRNAs that encode the NP and RdRp, respectively. Subsequently, replication of the full-length S and L vRNAs produces S and L anti-genome RNAs (agRNA), which act as templates for synthesis of mRNAs encoding glycoprotein precursor (GPC) and Z proteins^7,8^. The GPC is post-translationally cleaved to form a trimeric assembly of structural proteins glycoprotein-1 (GP-1), glycoprotein-2 (GP-2) and a stable signal peptide (SSP)^9^.

Although LCMV has been intensively studied from an immunological perspective, many molecular aspects of its replication cycle remain poorly understood. One such area is the dependence of viral multiplication on host cell components. While the catalogue of critical cellular factors is increasing, with recent additions to the list including components of the coat protein complex I (COPI) and adaptor protein complex 4 (AP-4)^10^ the chaperonin TCP-1 Ring Complex TRiC/CCT^11^ and sialomucin core protein 24 (CD164) as a secondary receptor for entry^12^, many gaps in our knowledge remain.

Here, we investigated the ability of LCMV to infect cells via intercellular connections rather than the canonical extracellular infection route that involves egress into the extracellular space and subsequent entry. Many viruses, including coronaviruses^13^, retroviruses^14–17^, alphaviruses^18^ and orthomyxoviruses^19^, have all been shown to utilise intercellular connections as a method of cell-to-cell spread. Possibly this mechanism represents an efficient means of transmission that does not require intact virion formation and allowing evasion of the host immune response^20^. One such class of connections are tunnelling nanotubes (TNTs), which are membranous tubular structures possessing a backbone rich in filamentous-actin (F-actin) that vary in diameter between 50 and 200 nm and can extend for distances of up to 100 μm^21^. TNT connecting A549 cells have previously been described in the literature, which contain both actin and tubulin, and require HGF/c-Met/β1-integrin signalling for cell-cell formation^22^. TNTs allow cellular connectivity and have been shown to permit the transfer of varied components between distant cells, including organelles, vesicles, signalling molecules, and ions^17^.

Using recombinant LCMV variants with engineered epitope tags, we showed the three major LCMV structural proteins, NP, GP-1 and Z, all colocalised within TNT-like structures during infection. Furthermore, fluorescent in situ hybridisation (FISH) showed these connections also contained LCMV genomic sense RNA. Taken together, these observations suggest LCMV may utilize such connections to allow intact virions or RNPs to pass between cells. Blocking the extracellular route of infection by adding the potent LCMV neutralising antibody M28 to supernatants during infection revealed almost half of all LCMV transmission events were by intercellular connections. Consistent with this, by blocking extracellular transmission using M28 alongside simultaneous disruption of TNT formation using pharmacological inhibition, LCMV transmission was reduced to background levels.

This is the first report of cell-to-cell infection via TNT-like connections for any species within the *Bunyaviricetes* class, which reveals arenavirus transmission is more complex than originally thought. This study furthers our understanding of how arenaviruses manipulate the host to establish infection, which may aid in the development of effective antiviral therapies.

## Results

### Observation of rLCMV-eGFP infection of cultured cells suggests transmission may involve direct cell-to-cell connectivity

**Fig. 1:**
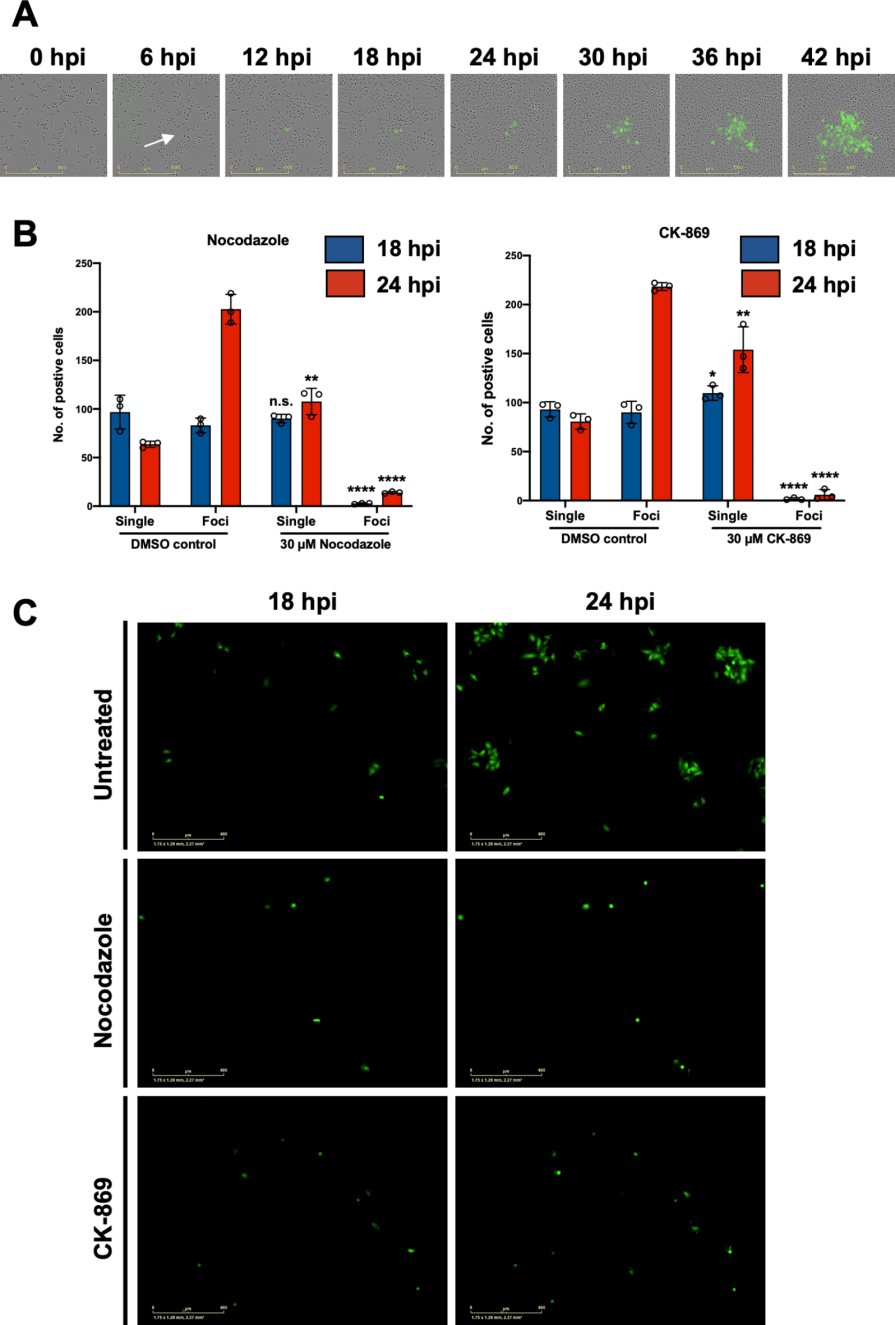
LCMV transmission within a culture involves cell-to-cell spread and is blocked by inhibitors of actin and tubulin cytoskeleton formation. (**A**) A549 cells were seeded and simultaneously infected with rLCMV-eGFP at a 0.001 multiplicity of infection (MOI). Phase and eGFP whole well images were taken every 6 h, and subsets cells monitored until 42 hpi. A single infected cell (white arrow) was monitored for the duration of the experiment. (**B**) A549 cells were infected with rLCMV-eGFP at an MOI of 0.01. At 3 hpi, 30 µM nocodazole or CK-869 was added to infected cultures and present for the duration of time analysed. Infected cells were counted at both 18 and 24 hpi and represented as single cells or multicellular foci. The average of three independent experimental repeats is shown (*n* = 3). Statistical analysis (T test) was performed for each inhibitor condition against the respective DMSO control. (**C**) Representative single image for untreated, nocodazole and CK-869-treated cultures, at both 18 and 24 hpi.

During routine LCMV infection of cultured cells, when omitting semi-solid media overlay, we noted that infected cells formed discrete foci rather than being widely dispersed throughout the culture. To further examine LCMV spread, cells were seeded and infected with previously described recombinant LCMV expressing eGFP (rLCMV-eGFP)^23^ at MOI 0.001, without semi-solid media overlay. Whole well live-cell fluorescent microscopy analysis was then used to continuously monitor infected cell clusters every 6 h post infection (hpi) (Fig. 1A). At 6 hpi, eGFP expression was first visualised within a single infected cell (Fig. 1A; white arrow). As infection progressed with time, an increasing number of neighbouring cells became infected, culminating at 42 hpi when a discrete fluorescent focus was visualised, with few cells outside of this showing evidence of infection. This finding suggested that LCMV spread within a culture may involve transmission via direct cell-to-cell contact.

### Disruption of cellular connections significantly attenuates LCMV cell-to-cell transmission

Previous studies have shown that transmission of influenza virus can occur via intercellular connections built around a rearranged cytoskeletal scaffold, and pharmacological disruption of these structures inhibits infectivity^19^. To test for the possible involvement of such structures in LCMV cell-to-cell transmission, A549 cells were^24,25^ infected with rLCMV-eGFP at an MOI of 0.01, after which non-toxic concentrations of either microtubule inhibitor nocodazole or F-actin polymerization inhibitor CK-869^24,25^ were added at 3 hpi (Fig. S1A), when internalization is known to be complete^23^. Transmission was assessed using live cell fluorescence microscopy to count multicellular foci, indicative of intercellular transmission, or single infected cells, indicative of spread involving extracellular egress (Fig. 1B and C). Visualization of DMSO-treated control cultures at 24 hpi revealed a high relative abundance of multi-cellular foci compared to single cells suggesting direct transmission between adjacent cells was a frequent event. In cells treated with either CK-869 or nocodazole, foci formation was significantly impaired at 24 hpi, with the most abundant infected cells detected as single entities, with few multicellular foci. The fold change reduction in foci formation was 14.5 for nocodazole and 36.5 for CK-869 treated cultures. To ensure this reduction in foci formation related to a block in cell-to-cell connectivity rather than overall virion production, we tested the extracellular titre from a single-round of infection in the presence of nocodazole and CK-869, and the observed effect was modest (Fig. S1B). Taken together, these data suggest that LCMV cell-to-cell transmission, and foci formation, depend on a functional cytoskeletal network, consistent with the involvement of intercellular connections.

### Cell-to-cell connections contain both actin and tubulin

**Fig. 2:**
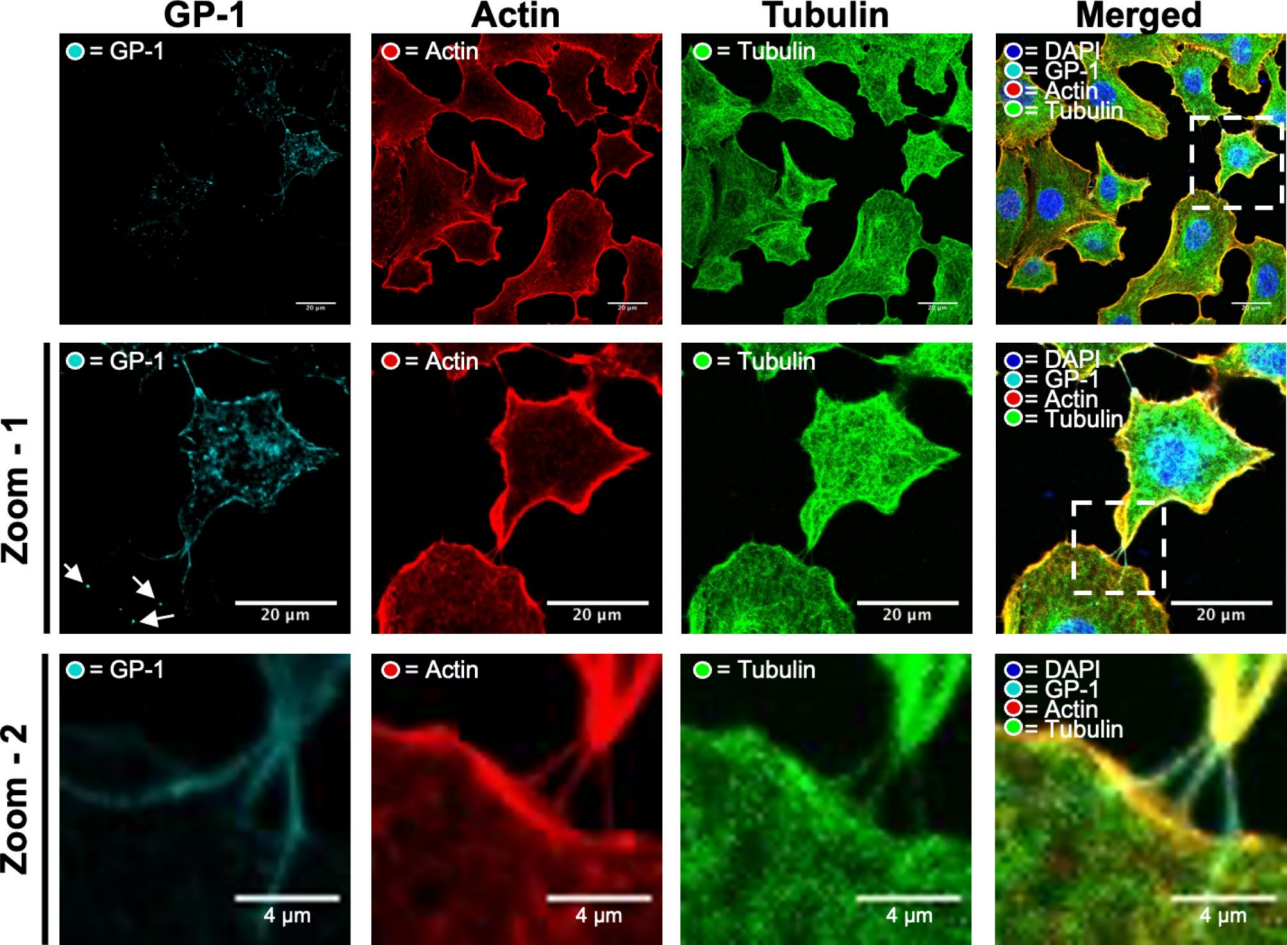
Intercellular connections between LCMV-GP-1-FLAG infected cells contain both F-actin and tubulin, as well as virion component GP-1. A549 cells were infected with rLCMV-GP1-FLAG at an MOI of 0.2. At 24 hpi, cells were fixed and then stained with DAPI and phalloidin (actin) alongside antisera specific for GP-1 (cyan) and a β-tubulin-specific Affimer (green), and then visualized by confocal microscopy. A magnified region of interest (white box in the central row) is shown on the middle row, zoom-1, where multiple cell-cell connections are visualized by staining with both tubulin and F-actin, shown further magnified in the bottom row, zoom-2. Additional points of interest are indicated by arrows to highlight punctate regions of LCMV GP-1 present in uninfected neighbouring cells, extending beyond F-actin and β-tubulin staining.

To further investigate the role of cell-to-cell contacts in LCMV spread, we first needed to visualize such connections, and confirm that viral components could be detected within. To do this, A549 cells were infected with our previously described rLCMV-GP1-FLAG^10^ expressing a FLAG tagged GP-1 and fixed at 24 hpi with staining to detect the cellular distribution of the LCMV GP-1 spike (cyan) alongside the cytoskeletal components F-actin (red) and β-tubulin (green), visualised using indirect immunofluorescence (IF) confocal microscopy. For clarity, two successive magnifications of intercellular connections are shown; zoom-1 (Fig. 2; middle row) and zoom-2 (Fig. 2; bottom row).

At 24 hpi, GP-1 was predominantly localised within perinuclear regions, but also within puncta throughout the cytosol and proximal to the plasma membrane (Fig. 2; zoom-1). As expected, inspection of the intercellular spaces revealed GP-1 was also present within filamentous projections that extended between adjacent cells (Fig. 2; zoom-1 and zoom-2). In addition, IF analysis revealed that both F-actin and β-tubulin were present within the tubular cell-to-cell connections. These cell-to-cell connections varied in size but often appeared to connect an infected cell containing abundant GP-1 to cells devoid of GP-1, likely uninfected. To view these connections more clearly, we routinely used phase-contrast imaging alongside IF confocal microscopy to reveal the boundary of the TNT-like structures, with further representative cell images shown in Fig. S2A. In many cases, multiple GP-1 puncta were observed extending beyond cell-to-cell connections into the cytosol of the uninfected cell (Fig. 2; zoom-1 white arrows), consistent with the connections being open-ended. As these intercellular connections share some of, but not all, the distinguishing characteristics of TNTs, namely rich in F-actin and open-endedness, hereafter we refer to these connections as ‘TNT-like’.

To understand if LCMV induced the formation of TNT-like connections, we quantified the total number of connections between 50 cell pairs for both uninfected and infected cultures (MOI 0.5), which revealed no significant difference in the total numbers in response to infection (Fig. S2B). These results suggest that LCMV infection does not induce the formation of TNT-like connections, but instead LCMV likely subverts existing connections for transmission.

### Visualization of intercellular connections within LCMV-infected cultures using stimulated emission depletion (STED) microscopy

To view intercellular connections with greater detail, we next observed A549 cells infected with rLCMV-GP1-FLAG using stimulated emission depletion (STED) microscopy, with staining for F-actin alongside LCMV spike component GP-1. This analysis revealed intercellular connections between cells to comprise a complex F-actin architecture, typically with multiple F-actin bundles running parallel with the long axis of the tubes, both bordering the tube as well as forming an internal central core.

**Fig. 3:**
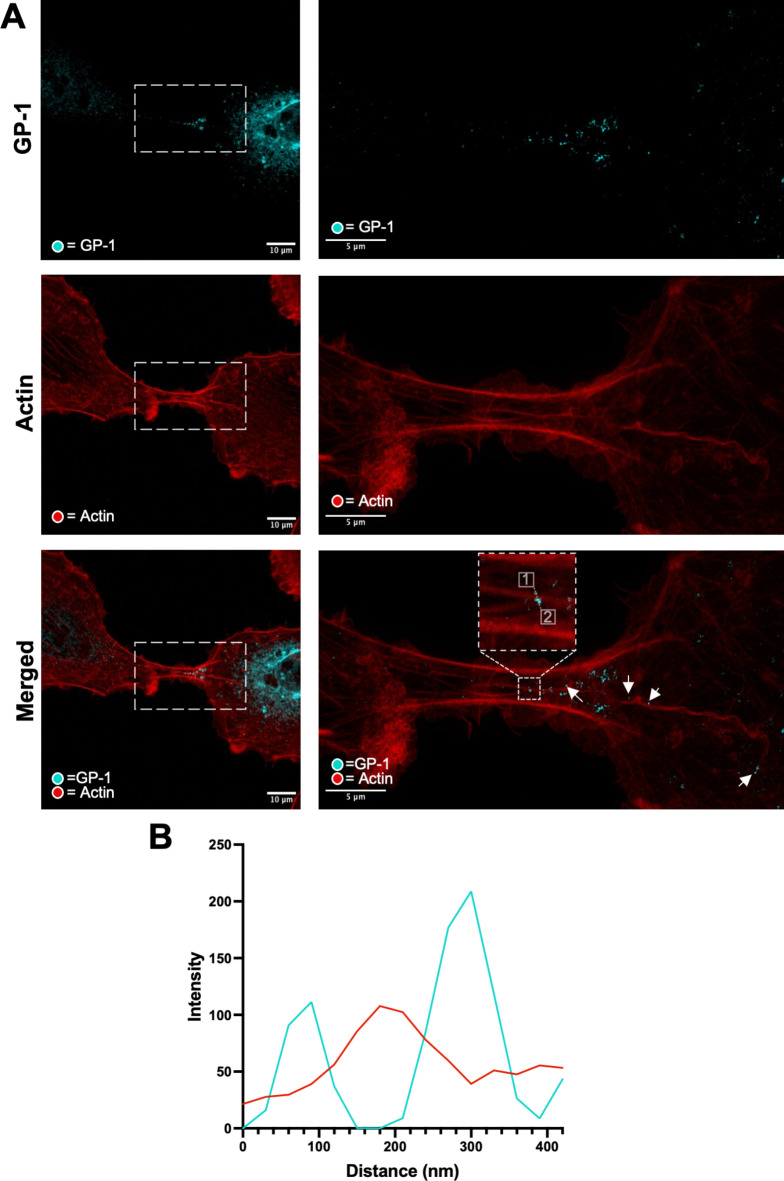
Stimulated emission depletion microscopy reveals the structural organisation of cell-to-cell connections between A549 cells infected with rLCMV-GP1-FLAG. (**A**) A549 cells were infected with rLCMV-GP1-FLAG at an MOI of 0.2. and at 24 hpi, cells were fixed, stained for GP-1-FLAG (cyan) and F-actin (red), then visualized by stimulated emission depletion microscopy (STED). An infected cell (left) and a zoom (right) region of interest (white box) are shown. Points of interest are indicated (arrows) to highlight punctate regions of LCMV GP-1 that exist along an F-actin branch. The bottom right panel is a magnified version of the region within the white box from the bottom left panel. (**B**) Line scan analysis of the region of interest in the zoomed merged image (white box), showing GP-1 peak intensities do not closely correspond to those of F-actin at this resolution.

In the image shown, one prominent F-actin filament emanated from within the cell on the right of the image and extend through the connection (Fig. 3A, bottom row arrows). The staining of GP-1 within the two interconnected cells was different; in the right-hand cell, GP-1 was widely-distributed with abundant intense signal suggestive of a late-stage infection, whereas in the left–hand cell, the GP-1 signal was of low intensity, suggestive of an early-stage infection. Within the cell-to-cell connection, the GP-1 signal exhibited a gradient of abundance, which decreased with distance away from the late-stage infected cell. The majority of GP-1 was located proximal to, but not coincident with, the central F-actin fibres (Fig. 3A, arrows). This observation was corroborated with line scan analysis (Fig. 3B; corresponding to dotted line in panel A [start 1, end 2]), which showed the peak signals corresponding to F-actin did not precisely overlap with GP-1, but instead were separated by approximately 90 nm. Interestingly, super resolution measurement of regions within the cell-to-cell connection confirmed GP-1 puncta were of a size (100–150 nm) expected for intact virions, but we cannot rule out the possibility that these objects are composed of GP-1 only. Taken together, these findings suggest that intercellular connections may act as conduits for the transport of LCMV components, or even intact virions, between neighbouring cells.

### Rescue of a recombinant LCMV variant with GP-1 FLAG-tag alongside Z HA-tag (rLCMV-GP1-FLAG-Z-HA)

**Fig. 4:**
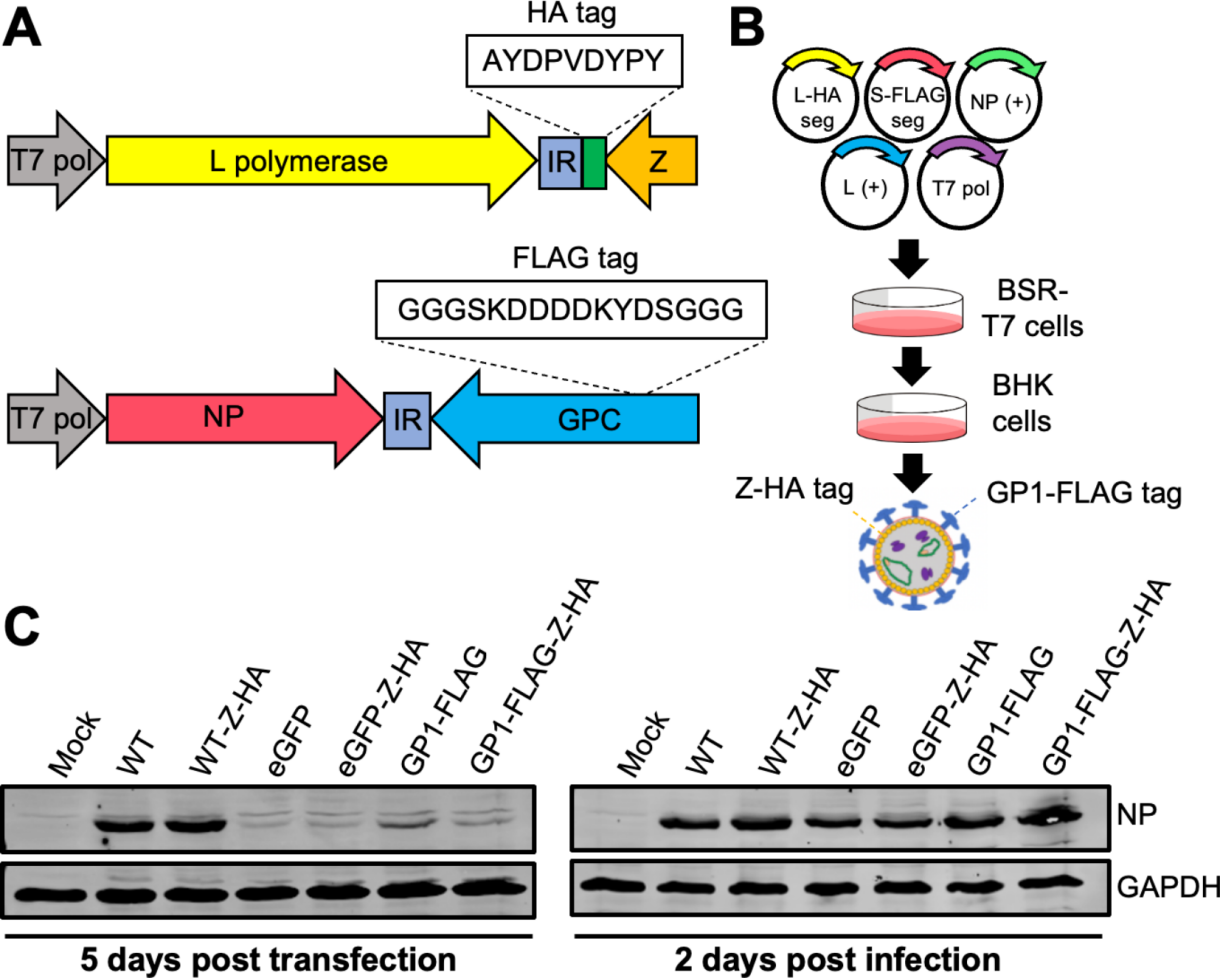
Rescue of a recombinant LCMV variant with GP-1 FLAG tag alongside Z HA tag (rLCMV-GP1-FLAG-Z-HA**).** (**A**) Schematic of LCMV S and L segments with respective tag insertions. (**B**) BSR-T7 cells were transfected with cDNAs expressing a LCMV S segment encoding a FLAG tagged GP-1 (S-FLAG segment), an L segment encoding a HA tagged Z (L-HA segment), as well as NP and L open reading frames (support plasmids, L + and NP+) and a plasmid expressing T7 RNA polymerase. At 5 days post transfection (5 dpt), cellular supernatants were transferred to BHK cells, and at 3 days post infection (3 dpi), cell lysates were analysed by western blotting using NP antisera, with viral supernatants harvested and subject to focus forming assays (FFA). (**C**) Western blot analysis of transfected BSR-T7 and infected BHK-21 cell cultures confirmed rLCMV-GP1-FLAG-Z-HA rescue (alongside all other mutants), using antisera specific for LCMV NP and GAPDH as loading control.

The results of the previous section showed that viral component GP-1 can travel through TNT-like connections. To investigate whether other viral components could similarly pass within TNT-like connections, we combined segments from two previously described infectious rLCMV variants, namely rLCMV-Z-HA^23^ and rLCMV-GP1-FLAG^10^, generating the rLCMV reassortant rLCMV-GP1-FLAG-Z-HA (Fig. 4A). For this virus, the S segment expressed FLAG-tagged GP-1 and the L segment expressed a C-terminally HA-tagged Z, allowing simultaneous detection of LCMV NP (using NP antisera), Z and GP-1, the three major structural components of the virion, within infected cells (Fig. 4B). Western blot analysis confirmed successful rescue of rLCMV-GP1-FLAG-Z-HA (Fig. 4C and Fig. S3), with the presence of LCMV NP at 5 days post transfection indicating successful recovery of rLCMV-GP1-FLAG-Z-HA. Subsequently, supernatants were transferred to fresh BHK cells and harvested at 2 days post infection (dpi). Western blot analysis revealed successful recovery of rLCMV-GP1-FLAG-Z-HA, and the relative NP abundance for all mutants recovered were comparable to WT (Fig. 4C). Subsequently, rLCMV-GP1-FLAG-Z-HA stocks were amplified and titrated in BHK cells, reaching a titre of 5 × 10^5^.

### LCMV GP-1, NP and Z puncta co-localise within TNTs during infection

**Fig. 5:**
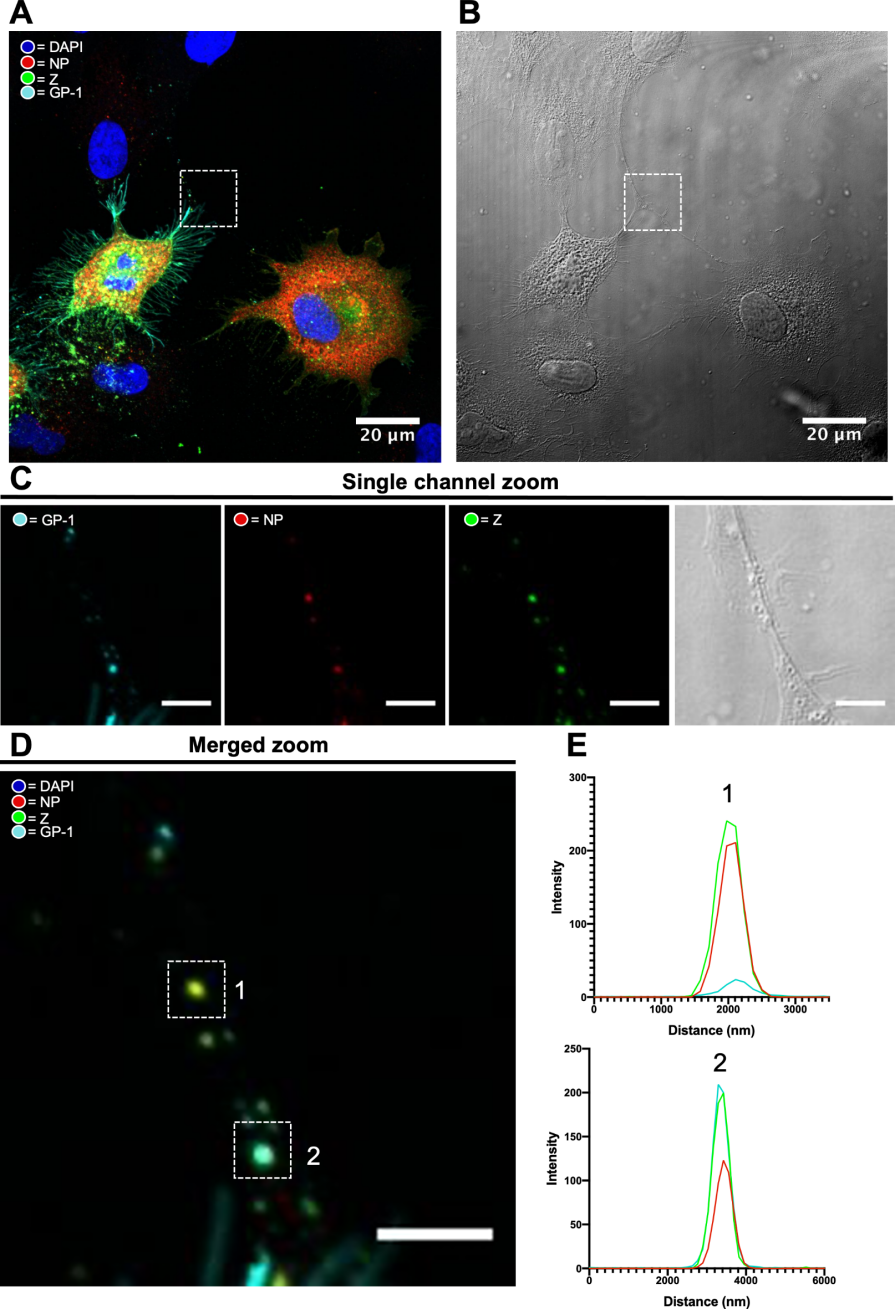
GP-1, NP and Z puncta co-localise within TNT-like connections during rLCMV-GP1-FLAG-Z-HA infection. (**A**) At 24 hpi A549 cells infected with rLCMV-GP1-FLAG-Z-HA at an MOI of 0.25 were fixed, stained for GP-1 (cyan), NP (red) and Z (green) and visualized by widefield microscopy, also imaged using phase contrast, shown in (**B**) alongside. (**C**) A TNT-like connection between an infected and uninfected cell is boxed in both widefield and phase images, shown magnified as separate channels with the same zoomed image shown with channels merged in (**D**). (**E**) Line scan analysis of the region of interest in the zoomed merged image showing alignment the intensity peaks for NP, GP-1 and Z.

Next, rLCMV-GP1-FLAG-Z-HA was used to assess the presence of NP, Z and GP-1 within infected cells and TNT-like connections. A549 cells were infected with rLCMV-GP1-FLAG-Z-HA at a MOI of 0.25, and at 24 hpi cells were fixed and the cellular localisation of NP (red), GP-1 (cyan) and Z (green) was assessed using specific antisera via widefield IF microscopy (Fig. 5).

At 24 hpi the distribution of each of NP, GP-1 and Z was distinctive. In agreement with our previous findings, LCMV NP formed discrete perinuclear puncta, as well as being widespread throughout the cytoplasm^23^. LCMV GP-1 localisation differed to that of NP, being predominantly punctate and perinuclear as well as being abundantly distributed along the plasma membrane. Interestingly, Z showed a similar cellular distribution to GP-1, with clear and abundant Z/GP-1 co-localisation in perinuclear regions, and co-localization at discrete locations proximal to the plasma membrane, possibly representing virus assembly sites.

In addition, multiple puncta were observed within TNT-like connections between LCMV infected and uninfected cells (Fig. 5A; white boxes in top row) containing NP, GP-1 and Z. Line scan analysis across two discrete puncta (Fig. 5A; white boxes in bottom row) showed the peak intensities for NP, GP-1 and Z viral components were spatially closely aligned (Fig. 5B) although interestingly, the relative composition of these three proteins within the puncta was not consistent. Taken together, these observations show that in addition to the GP-1 envelope spike protein, internal virion components NP and Z are also able to pass through TNT-like connections as discrete punctate objects. These observations raise the interesting possibility that assemblies of multiple viral proteins, or even intact virions, may pass between cells through intercellular connections.

### LCMV NP and viral genomes co-localize within TNT-like connections

For infectivity to transmit between cells via intercellular connections, we reasoned these connections must contain, at minimum, the LCMV genome enwrapped in NP to form the RNP. To test this, we performed fluorescent in situ hybridisation (FISH) using probes designed to detect negative sense LCMV vRNA genomes, which represents the LCMV genetic material found within infecting virions.

To achieve this, we generated a set of fluorescently labelled probes specific for the NP coding region of the negative sense rLCMV S RNA segment (list of probes; supplementary dataset S1), with which we performed FISH on A549 cells infected with rLCMV at an MOI of 1.0 at 24 hpi using wide-field microscopy. To specifically detect assembled LCMV RNPs, which represent the form in which vRNAs are packaged within virions, cells were also co-stained with antisera specific for LCMV NP.

**Fig. 6:**
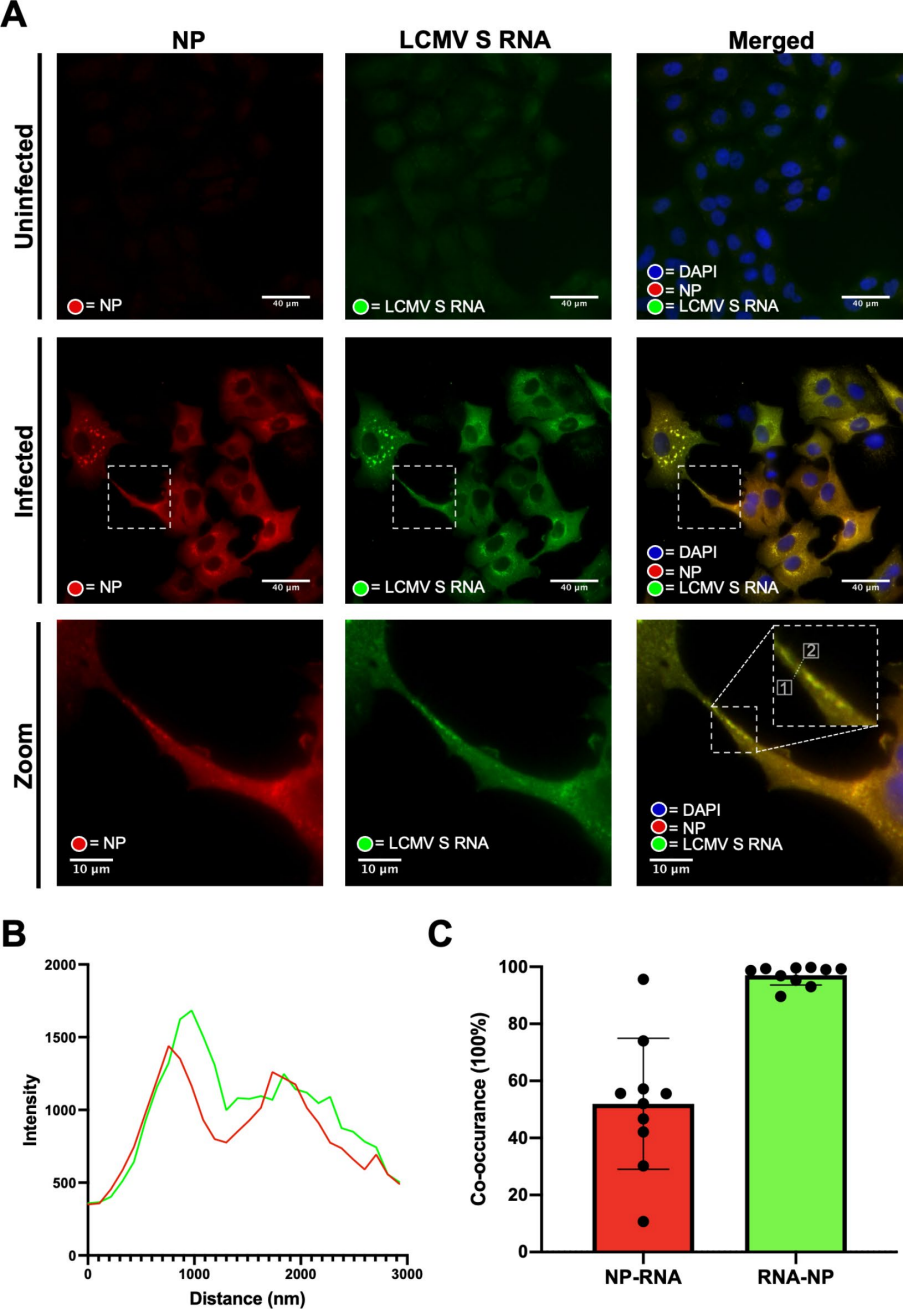
LCMV vRNA is present alongside NP in TNT-like connections. (**A**) At 24 hpi rLCMV infected A549 cells at an MOI of 1 were fixed and then stained for NP (red). Subsequently, coverslips were hybridised overnight with 48 specific FISH probes designed to hybridize with LCMV S vRNA (green). Widefield microscopy was then utilised to visualise NP and S vRNA within TNT-cell connections. Cell nuclei were DAPI stained. Uninfected cells and infected cells are shown. A zoomed-in region of interest (white box in the central row) is shown on the bottom row. (**B**) Line scan analysis of the region of interest in the zoomed merged image (dashed line in the bottom right panel of (**A**)) revealed peaks in intensity of NP and S vRNA were similar. (**C**) Co-occurrence analysis using the Manders coefficient method was performed for 10 TNT-like connections. The percentage of LCMV NP to S vRNA (Red) and S vRNA to LCMV NP (green) is shown, and individual images plotted.

As anticipated for an MOI of 1.0 at this time point, most cells appeared infected as evidenced by staining by FISH and NP (Fig. 6A, middle row). Within cells, NP distribution was consistent with previous findings, and FISH analysis revealed close co-localisation of NP and LCMV S segment vRNA at several locations within the infected cells (Fig. 6A, middle bottom rows; zoom) sometimes in perinuclear locations, but also as dense puncta throughout the cytosol. Within TNT-like connections, signals corresponding to NP and S segment vRNA were visualised (Fig. 6A, white boxes in middle and bottom rows) as discrete puncta, within which NP and RNA signals appeared to closely co-localize. This was corroborated using line scan analysis, which revealed peaks corresponding to both NP and S vRNA were closely coincident (Fig. 6B; corresponding to the dotted line in panel A, bottom row; start 1, end 2). Such punctate objects were common within TNT-like structures and further representatives are shown, along with corresponding line scans, in Fig. S4. The proximity of the LCMV NP and S segment vRNA signals was consistent with their location within the assembled RNP, with the small offset in peak spatial intensity likely a reflection of the differing approaches used for target detection; the FISH probes bind directly to the S vRNA target and emit signal from fluorogenic probe-bound dyes. In contrast, the NP signal results from binding of primary and fluorescent secondary antibodies, thus some spatial separation between target and fluorophore was expected. To quantify the extent of S vRNA and LCMV NP co-localisation within connections, we performed co-occurrence analysis of 10 different TNT-like tubes (Fig. 6C), which revealed an RNA/NP mean co-occurrence of 97%, consistent with the presence of RNPs. In contrast, mean co-occurrence of NP/RNA was 52%, suggesting some NP may also be trafficked through connections in an RNA-free state.

Taken together, the results of this analysis are consistent with a scenario in which LCMV RNP components, namely vRNA and NP, can travel within TNT-like connections between cells. These RNPs likely have the capacity to initiate infection, as has recently been shown for the related segmented negative sense RNA virus, influenza A virus (IAV)^19^.

### Blocking extracellular LCMV transmission with a potent neutralising antibody reveals that LCMV utilises cell-to-cell spread during infection

**Fig. 7:**
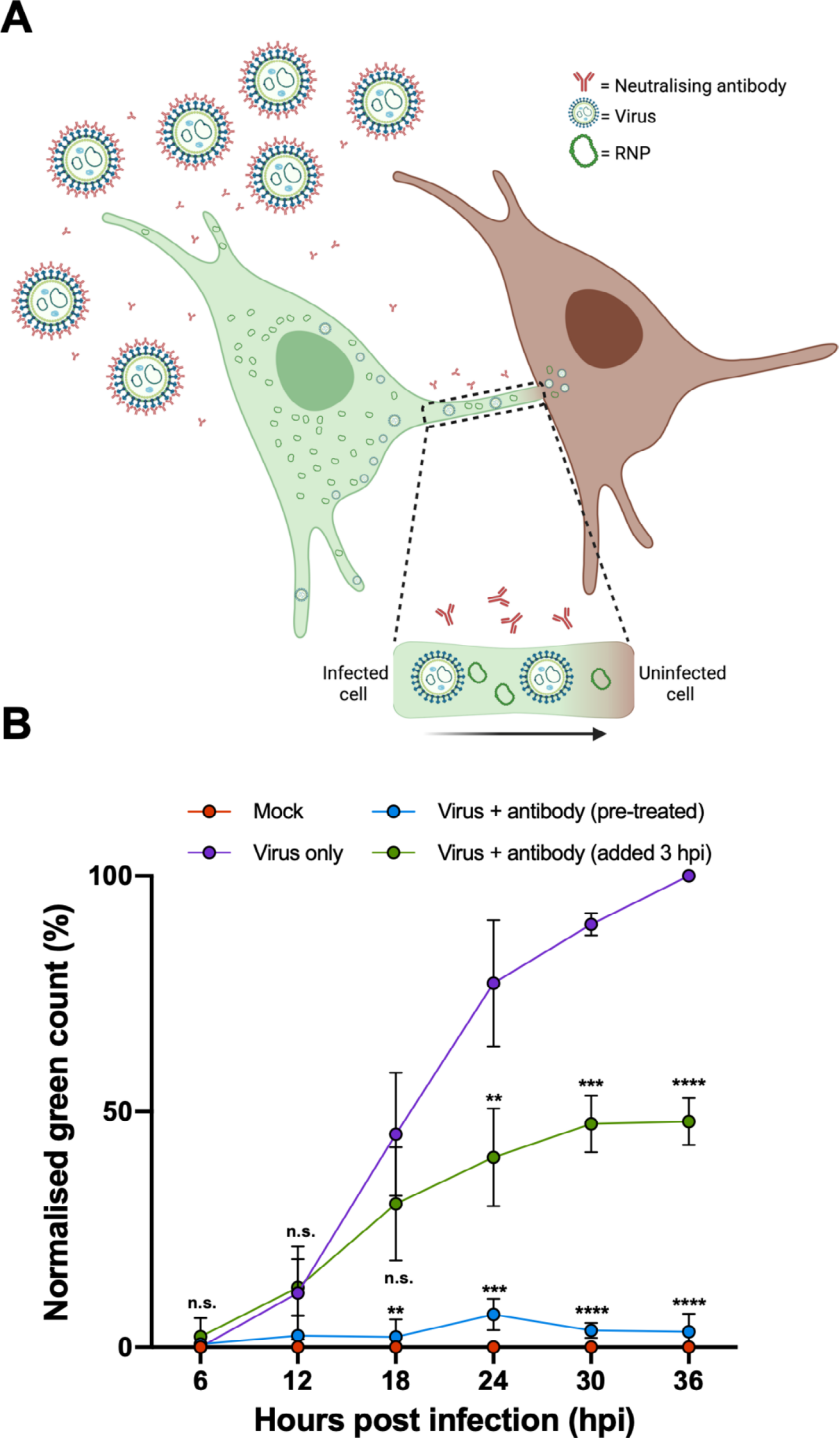
LCMV can efficiently spread through a culture despite blocking extracellular transmission using a potent neutralising antibody. (**A**) Schematic representation of the model for intercellular LCMV transmission in the presence of potent neutralizing antibody M28 that targets GP-2. Virus released via the canonical plasma membrane egress mechanism are rendered non-infectious through M28 binding the GP-2 portion of the spike complex. Only virions or RNPs that pass-through TNT-like connections can transmit infectivity to new cells. Image created with Biorender. (**B**) A549 cells were infected with rLCMV-eGFP at an MOI of 0.1 that was either pre-treated with antibody M28 (blue plotted points; 5 µg/mL antibody for 1 h) or virus only (in the absence of antibody; purple plotted points). For virus + antibody (green plotted points), 5 µg/mL antibody was added to cultures at 3 hpi, following LCMV entry and uncoating, which remained for the duration of infection. The total green count (number of green cells) was measured every 6 h and normalised against virus only (purple) cultures at 36 hpi. The average of three independent experimental repeats is shown (*n* = 3), with error bars showing standard deviation at each time point. Statistical analysis (T test) was performed for each condition against the respective virus only control timepoint.

To confirm that LCMV infectivity can pass between cells via TNT-like connections, we made use of a recently described LCMV neutralising antibody (M28) that targets GP-2^26^. We reasoned that when added to infected cell supernatants, this antibody would block LCMV infection via the extracellular route but would not hinder cell-to-cell transmission via intercellular connections (Fig. 7A).

First, to confirm the potency of this neutralising antibody under the selected experimental conditions, M28 at 5 µg/mL was pre-incubated with rLCMV-eGFP prior to infection of A549 cultures. To measure virus transmission throughout the culture the total green cell count (TGC) was determined at successive 6 hpi time points up to 36 hpi, at which time a TCG value of just 3% was recorded (Fig. 7B; blue plotted points) and normalized to that of the virus only (in the absence of antibody) control (Fig. 7B; purple plotted points). This was statistically indistinguishable from the TCG of mock infected cultures (Fig. 7B; red plotted points) and thus confirmed that the antibody effectively blocked extracellular transmission in the culture to background levels of detection.

Next, to investigate whether rLCMV-eGFP could spread within the culture when extracellular transmission was blocked, cultures were infected with rLCMV-eGFP at an MOI of 0.1, with M28 added at 3 hpi, at which time virus entry is complete but virion assembly has not yet commenced^23^. This protocol would allow virus to enter these initial cells, but the presence of antibody in the media thereafter would block any subsequent transmission by the extracellular route. To further demonstrate that M28 was able to neutralize any viruses that might be released, supernatants of rLCMV-eGFP infected cells were collected at 18 hpi, treated with 5 µg/mL antibody and titrated via focus forming assays (Fig. S5), which revealed no infectivity.

Blocking extracellular transmission using these experimental conditions resulted in a TCG value of 47% at 36 hpi (Fig. 7B; green plotted points) when normalized to the virus only control. This showed that significant transmission had occurred throughout the culture despite the continued presence of potent neutralizing antibody.

Taken together, these results show that a major fraction of LCMV transmission within a culture occurs without involvement of the extracellular space, and the detection of LCMV components NP, Z, GP-1 and vRNA within TNT-like connections, strongly suggests these structures represent effective conduits of LCMV infectivity.

## Discussion

Curious as to why LCMV spread throughout cultured cells appeared to be spatially restrained thus forming infected cell foci, we reasoned that infectivity may pass between cells by TNT-like intercellular connections. To investigate this, we first used epitope tagged LCMV variants rLCMV-GP1 and rLCMV-GP1-FLAG-Z-HA to confirm that viral proteins GP-1, Z and NP were present within these connections, colocalized within discrete punctate objects. Furthermore, FISH analysis showed these TNT-like connections also contained LCMV S genomic vRNA in close proximity to NP, indicative of RNP structures that would be expected to be found within infectious virions, or that could themselves represent the infecting entity. Although LCMV NP alone has previously been detected between cells^27^, our current study is the first to reveal the co-localization of all major LCMV structural components namely NP, genomic RNA, Z and GP1 as discrete puncta within intercellular connections. Furthermore, we showed LCMV could transmit within a culture in the presence of a potent neutralising antibody, which effectively blocked extracellular transmission. This revealed that LCMV transmission could occur without involvement of the extracellular space and that cell-to-cell LCMV transmission may represent up to around 50% of total spread during infection. Taken together, our data suggests that LCMV infectivity can traffic between cells through intercellular connections, and this mode of virus spread represents a significant proportion of total virus transmission during infection.

This work is the first report describing cell-to-cell transmission via TNT-like connections for any species of the *Bunyaviricetes* class. Nevertheless, many other viruses have recently been revealed to rely on TNT or TNT-like structures during infection including species of retroviruses, herpesviruses, alphaviruses, pneumoviruses, orthomyxoviruses, paramyxoviruses, flaviviruses and picornaviruses^28^.

Within the *Coronaviridae* family, SARS-CoV-2 virions have recently been visualised to ‘surf’ on the outside of TNTs, connecting cells during infection, along with evidence to suggest mature virions are also present within the interior of the same structures^13^. Furthermore, this study demonstrated transmission of SARS-CoV-2 between permissive and non-permissive neuronal cells via TNT connections. Thus, TNT connections could potentially act as a mechanism for widespread dissemination of viruses within a host, circumventing different cellular or tissue-type restrictions, such as the requirement for specific entry receptors, allowing spread to tissues and organs distinct from the initially infected target cells.

The reliance of SARS-CoV-2 on TNTs was further investigated with the use of neutralising antibody to block extracellular transmission of a pseudotyped lentivirus, resulting in no significant decrease in cell-cell spread^29^. Thus, it was proposed that cell-to-cell TNT connections may represent an efficient mode of transmission, allowing evasion of host immune cell responses. It will be interesting to test whether LCMV also exploits TNT-like structures during infection of target host tissues, and also, whether this mode of transmission occurs during infection of intact organisms. Since LCMV has been extensively studied from the perspective of immune evasion and the balance between chronic or acute infections^30^ it would be interesting to investigate whether cell-to-cell transmission plays a significant role in these processes. In addition, a greater understanding of how LCMV propagates in the natural host, where multiple cell types are susceptible to infection, may help explain how LCMV is able to grow to high titres in suspension cultures^31^, which may preclude the formation of cell-to-cell connections.

Interestingly, IAV, which like LCMV is an enveloped negative-sense segmented RNA virus, has been shown to utilise TNT connections for spread during infection. However, for IAV the infectious entity is not proposed to be a fully assembled mature virion, but instead is believed to comprise solely the RNP, devoid of both matrix and the spike-embedded envelope^19,22^. Rab11a is known to be involved in transport of RNPs to assembly sites at the plasma membrane for subsequent budding and transmission involving extracellular release, and Rab11 was also shown to play a role in movement of IAV vRNPs across TNT connections^19^. Interestingly, analysis of IAV infection outcome revealed the movement of IAV segments through TNT connections acted to facilitate genome reassortment during co-infections, with important consequences for virus evolution and possible emergence of pathogenic variants^19^.

For LCMV, we are interested in determining the composition of the intercellular infecting entity. While our data shows that the internal RNP as well as Z and GPC components are colocalised within TNT-like connections, we are unable to determine whether the RNP alone, or the RNP in combination with one or more of the matrix or envelope components are required to transmit infectivity. What is probable, and consistent with our data, is that both intact virions and non-enveloped RNPs can traffic through TNT-like connections. Further investigations are required to understand if only one or both options results in productive infection within subsequent cells, but clearly, the distinction between these two possibilities has implications for infection of the connected cell. If the transmitting object is an RNP, then presumably this would be able to initiate infection in the same way as if just released from an endocytic vesicle. In contrast, if the transmitting object is an intact enveloped virion, it must follow an infection pathway in the new cells that allows virion disassembly and RNP release by interaction with its secondary receptor CD164, that is specifically located within late-endosomal compartments, and how this could be achieved is unclear. It is interesting to note that as arenaviruses assemble at the plasma membrane, non-enveloped RNPs must necessarily transit across the cytosol from their replication site, as occurs for IAV, where they acquire their envelope, matrix and spike complex. This allows the possibility that non-enveloped RNPs may be substrates for cytoskeletal trafficking pathways that lead to the plasma membrane, but alternatively, these cytoskeletal pathways may also lead through intercellular connections delivering RNPs to new cells. If intact virions are indeed capable of passing through cell-to-cell connections, it is intriguing to speculate how such particles can be generated, given the plasma membrane is the site of virion assembly. One possibility is that some cell-to-cell connections are not open-ended tubes, but instead possess membranous structures at one or both ends that could potentially allow formation and subsequent passage of an enveloped virion into the interior of the connecting structure. Such closed tubes have been described alongside open-ended tubes^32^.

Further investigations are required to understand if the movement of virus components within TNT-like connections is an active or passive process, or if there is a reliance on host cell components for this transport, such as Rab11, as described for IAV, above^19^. Interestingly, STED analysis of rLCMV infected cells stained for GP-1 and F-actin (Fig. 2B) showed the corresponding signals were proximal but not coincident. This observation raises the possibility that F-actin associated proteins such as the resident myosin motors may play a role. The host cell motor protein myosin II has been implicated in ‘viral surfing’ for several viruses including murine leukaemia virus, avian sarcoma leukosis virus and vesicular stomatitis virus^33^. In the context of HIV infection, host cell motor protein non-muscle myosin II (NMMII), has been implicated in the movement of Gag and Env through cell-cell TNTs during infection^16^. It is unclear if LCMV requires the involvement of any cellular motors, or if an association with growing F-actin fibres is sufficient for TNT trafficking, and experiments to investigate this are underway.

Developing a clear understanding of transmission routes is important in order to formulate antiviral strategies. The role of cell-cell transmission of hepatitis C virus (HCV) for evasion of neutralising antibodies is well understood^34^. Direct acting antivirals (DAAs) are effective therapeutics for HCV, yet resistance can occur. In a recent study, cell-cell transmission was shown to be the primary route of HCV DAA-resistance, leading to infection persistence. By targeting cell-cell transmission alongside DAA treatment, resistance was overcome, and HCV was again eliminated in a cell culture model^34^.

Overall, this study reveals that a major fraction of LCMV transmission within cultured cells occurs via intercellular transmission, without involvement of the extracellular space. It will be interesting to examine whether other arenaviruses from both OW and NW clades utilise TNT-like connections as transmission conduits, and also to test whether these connections are formed in the context of intact tissues with an infected host.

## Materials and methods

### Plasmid design and virus rescue

Construction of plasmids expressing S and L segments for rescue of rLCMV-WT, rLCMV-eGFP, LCMV-Z-HA^23^ and LCMV-GP1-FLAG^10^ have been previously described, along with corresponding rescue protocols. Rescue of rLCMV-GP1-FLAG-Z-HA was achieved using these same protocols, by transfection of BSR-T7 cells with cDNAs expressing the S segment of rLCMV-GP1-FLAG along with the L segment of rLCMV-Z-HA (Fig. 4). Also transfected were supporting plasmids expressing the ORFs of LCMV NP and LCMV L proteins, as well as bacteriophage T7 RNA polymerase. At 5 days post transfection (dpt), cell supernatants were transferred to BHK cells allowing amplification of any rescued viruses. At 2 days post infection (dpi), viral supernatants were collected and subject to focus forming assays (FFA). Titred viral stocks gained were utilised for bulking in BHK cells at an MOI of 0.001, and subsequently harvested at 3 dpi.

### Virus infections

To generate virus stocks, T175 flasks of BHK cells seeded at 5 × 10^6^ the day prior were infected at an MOI 0.001. At 3 days post infection, viral supernatant was harvested and centrifuged to remove cell debris (x4000 g), aliquoted (80 µL) and frozen for subsequent viral titration. For all other viral infections, cell monolayers were infected with LCMV at the specified MOI in either serum-free (SFM), 2.5% or 10% FBS DMEM, depending on cellular requirements, at 37 °C. After 1 h, the inoculum was removed and SFM, fresh 2.5% or 10% DMEM was then applied for the duration of the infection. For synchronised infections, LCMV was bound on ice to cells for 1 h. Subsequently, the inoculum was removed, monolayers washed x3 with PBS and SFM, fresh 2.5% or 10% DMEM was then applied for the duration of the infection.

### Viral titration

Determination of virus titres was achieved through focus forming assays (FFA). Viral stocks requiring titration were serially diluted in SFM to infect fresh monolayers of BHK cells seeded at 1 × 10^5^ in a 24 well-plate. After infection, medium containing virus was removed and 1 mL 1:1 ratio of 10% FBS DMEM to 1.6% methylcellulose was reapplied, and the cells incubated for a further 3 days. For rLCMV-eGFP titration, the Incucyte Zoom S3 live cell imaging system (Sartorius) was used to image whole wells and detect fluorescent rLCMV-eGFP foci, which were then counted for titre determination. For rLCMV-WT titration, cells are fixed using 4% (vol/vol) paraformaldehyde (PFA) for 15 min and washed three times with PBS. Cells were then incubated with permeabilisation buffer (0.3% [vol/vol] Triton X-100, 2% [wt/vol] FBS in 1 x PBS) for a further 10 min, and then washed three times with PBS. Following this, cells were incubated with 1 mL blocking buffer (1% [wt/vol] BSA in PBS) for 1 h. The cells were then incubated for 1 h with 150 µL/well LCMV NP primary antibody (generated in-house, 1:1000 in blocking buffer), and washed three times with PBS. Following this, cells were incubated for 1 h with 594 Alexa Fluor secondary antibody (Life Technologies; 1:500 in blocking buffer), and subsequently washed four times with PBS. The Incucyte Zoom S3 live cell imaging system was then used to image whole wells of the plate to detect red rLCMV-WT focus forming plaques. Plaques were quantified, and virus titres determined. For rLCMV-FLAG titration, the protocol for rLCMV-WT was followed, yet cells were incubated for 1 h with 150 µL/well LCMV NP (in-house, 1:1000 in blocking buffer) and FLAG (Sigma, 1:500 in blocking buffer) primary antibodies. Following this, cells were incubated for 1 h with 488 (FLAG) and 594 (LCMV NP) Alexa Fluor antibodies (Life Technologies; 1:500 in blocking buffer), then washed four times with PBS.

### Analysis of LCMV infection progression

Trypsinised A549 cells were seeded in a 12-well plate at 1 × 10^5^ cells/well in 1 mL 10% FBS DMEM; simultaneously, cells were infected with rLCMV-eGFP, at an MOI of 0.001. To investigate spread throughout a culture, whole well images were taken at 6 h intervals using an Incucyte Zoom S3 live cell imaging system (Sartorius).

### Immunofluorescence (widefield and confocal microscopy)

Trypsinised A549 cells were seeded onto a 19-mm round glass coverslips (VWR) in a 12-well plate at 1 × 10^5^ cells/well, followed by incubation at 37 °C. At 24 hpi, 1 mL 4% (vol/vol) paraformaldehyde in PBS was added directly on top of the 1 mL infection media for 20 min at room temperature. After fixation, the cells were washed three times in PBS and then incubated in permeabilisation buffer (0.3% [vol/vol] Triton X-100, 1% [wt/vol] bovine serum albumin [BSA] in PBS) for 10 min at room temperature. Following permeabilisation, the monolayers were washed three times with PBS and incubated with blocking buffer (1% [wt/vol] BSA in PBS) for 1 h. Subsequently, primary antibody made in the BSA blocking buffer containing NP (1:500, in house [sheep]), FLAG (1:100 [mouse/rabbit]) and HA (1:500 [mouse/rabbit]) was incubated for 1 h at room temperature. The cells were then washed three times with PBS and incubated with corresponding Alexa Fluor 488, 594 and 647 secondary antibodies (Life Technologies; 1:500 in BSA blocking buffer) for 1 h at room temperature in a light protected vessel. Cell monolayers were then washed four times with PBS before addition of appropriate stain/dye; F-actin (Phalloidins, Texas Red, 5 µL/well in 500 µL blocking buffer) and β-tubulin (affimer; 1:200; A gift from Professor Michelle Peckham, University of Leeds, UK), for 1 h in a light protected vessel. Cell monolayers were then washed four times with PBS and mounted onto glass coverslips with the addition of Prolong Gold Antifade reagent with DAPI (Thermo Fisher Scientific), cured, sealed, and stored at 4 °C. Images were then taken on either Zeiss LSM 880 confocal microscopy or Olympus Widefield Deconvolution Microscope and processed using Zen (Blue Edition) software and Fiji (Image J). Line scan analysis was performed utilising Zen (Blue Edition).

### TNT-like connection inhibition utilising nocodazole and CK-869

Trypsinised A549 cells were seeded in a 12-well plate at 1 × 10^5^ cells/well in 1 mL 10% FBS DMEM and incubated for 16- to 24- h. Cells were subsequently infected with 500 µL rLCMV-eGFP at an MOI of 0.01, and at 3 hpi 500 µL of nocodazole (60 µM) or CK-869 (60 µM) was added to give a 30 µM final concentration. To investigate spread throughout a culture, 16-images were taken for each condition at 18- and 24- hpi using an Incucyte Zoom S3 live cell imaging system (Sartorius). For each condition, at both 18- and 24 hpi, infected cells were manually counted and represented as single or foci (defined as a minimum of two infected adjacent cells). The average of three independent experimental repeats is shown, with error bars showing standard deviation at each time point (*n* = 3). To ensure that the major effect of nocodazole and CK-869 was on cell-to-cell connections, viral egress was analysed after a single round of infection. A549 cells were infected at an MOI of 1 and infection incubated for 1 h at 37 °C. Subsequently, infection medium was removed and cells washed with 1 x PBS four times prior to replacement with 1 mL 10% FBS DMEM. At 3 hpi, nocodazole or CK-869 (30 µM) was added to the cultures. At 12 hpi, viral supernatants were collected and subject to focus forming assay analysis, and results expressed as normalised untreated titres. The average of three independent experimental repeats is shown, with error bars showing standard deviation at each time point (*n* = 3).

### Immunofluorescence (FISH)

FISH probes used were identical to a set previously described^35^ and synthesised by Stellaris. The protocol was followed according to manufacturer’s instructions, with probes hybridized overnight at 37 °C. As recommended by the manufacturer, FISH probes were visualized using widefield microscopy.

### Immunofluorescence (STED)

Trypsinised A549 cells were seeded onto a 19-mm round glass coverslip (VWR) in a 12-well plate at 1 × 10^5^ cells/well, followed by incubation at 37 °C. At 24 hpi, 1 mL 4% (vol/vol) paraformaldehyde in PBS was added directly on top of the 1 mL infection media for 20 min at room temperature. After fixation, the cells were washed three times in PBS and the incubated in permeabilisation buffer (0.3% [vol/vol] Triton X-100, 1% [wt/vol] bovine serum albumin [BSA] in PBS) for 10 min at room temperature. Following permeabilisation, the monolayers were washed three times with PBS and incubated with blocking buffer (1% [wt/vol] BSA in PBS) for 1 h. Subsequently, primary antibody in BSA blocking buffer containing FLAG, (1:100 [rabbit/mouse]) and HA (1:500 [rabbit/mouse]) was incubated for 1 h at room temperature. The cells were then washed three times with PBS and incubated with Fluro 647 secondary antibodies (Abberior STAR red; 1:500 in BSA blocking buffer) for 1 h at room temperature in a light protected vessel. Cell monolayers were then washed four times with PBS before addition of F-actin stain (Phalloidins, Texas Red, 5 µL/well in 500 µL blocking buffer) for 1 h in a light protected vessel. Cell monolayers were then washed four times with PBS and mounted onto glass coverslips with the addition of Prolong Gold Antifade reagent (Thermo Fisher Scientific), cured, sealed, and stored at 4 °C.

### Neutralising antibody assay

Trypsinised A549 cells were seeded in a 12-well plate at 1 × 10^5^ cells/well, followed by incubation at 37 °C for 16–24 h. For pre-treated condition, rLCMV-eGFP (MOI of 0.2) was incubated with 1 mL neutralising antibody (5µg/mL; made in 10% FBS DMEM) for 1 h. Remaining conditions (mock, virus only, virus + antibody [added at 3 hpi]) were subject to incubation with 1 mL 10% FBS DMEM for 1 h. Subsequently, the total 1mL for each condition was added to A549s, and infection allowed to progress. At 3hpi, 5µg/mL neutralising antibody was added inhibiting extracellular viral spread. Total green cell count was measured every 6 h using an Incucyte Zoom S3 live cell imaging system (Sartorius) and normalised for confluency variation. The average of three independent experimental repeats is shown, with error bars showing standard deviation at each time point (*n* = 3). To confirm the effectiveness of antibody M28 in neutralization of any newly released viruses, supernatants were collected for 18 hpi, and then subject to treatment with 5µg/mL neutralising antibody for 1 h. Subsequently, virus with and without neutralising antibody were subject to focus forming assays and titres expressed as normalised virus titres. The average of three independent experimental repeats is shown, with error bars showing standard deviation at each time point (*n* = 3).

## Electronic supplementary material

Below is the link to the electronic supplementary material.

Supplementary Material 1

Supplementary Material 2

## Data Availability

The datasets used and/or analysed during the current study available from the corresponding author on reasonable request.
