# Supplementary material for: Lymphocytic choriomeningitis arenavirus utilises intercellular connections for cell to cell spread
Source: Sci Rep. 2024 Nov 22;14:28961. doi: 10.1038/s41598-024-79397-w (PMC11584850; doi:10.1038/s41598-024-79397-w)
Supplement: Supplementary file 2 — Supplementary Material 2 [file 41598_2024_79397_MOESM2_ESM.pdf]

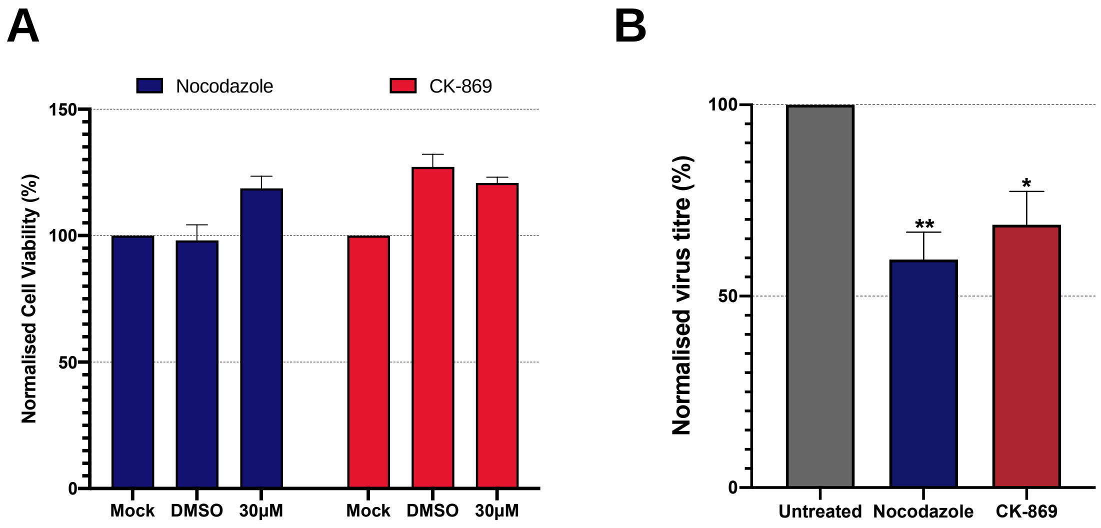

Figure S1. Cell viability and extracellular titres of A549 cells with TNT-like connection inhibitors. Analysis of 30 µM nocodazole (blue) and 30 µM CK-869 (red) toxicity in A594 cells. We performed MTT assay, normalised to mock untreated cells; the average of three independent experiments is represented (n=3), with error bars showing standard deviation. (B) Analysis of extracellular titre following treatment 30 µM nocodazole (blue) and 30 µM CK-869 (red) in A549 cells. Cells were infected with LCMV-eGFP at an MOI of 1, and at 3 hpi CK-869 and nocodazole were added to cultures. Viral supernatants were harvested following first virus release (12 hpi), and focus forming assays performed. The average of three independent experimental repeats is shown (n=3). Statistical analysis (T test) was performed for each inhibitor condition against the untreated control.

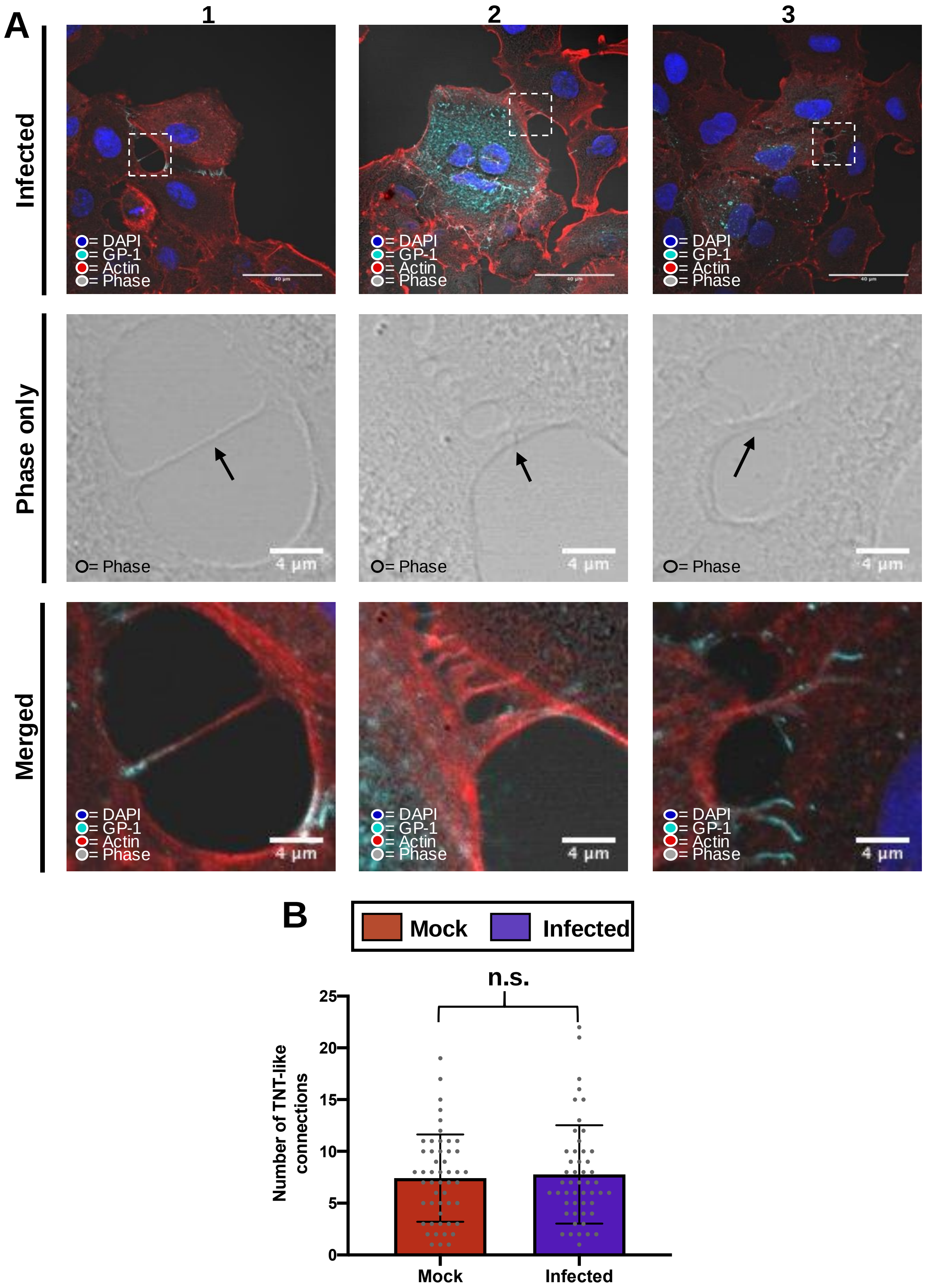

Figure S2. Phase-contrast microscopy of TNT-like connections during LCMV infection. A) A549 cells were infected with rLCMV-GP1-FLAG at an MOI of 0.2. At 24 hpi, cells were fixed and then stained for GP-1 (cyan) and F-actin (red). Confocal microscopy was then utilised to visualise GP-1 and F-actin for TNT-like structures connecting cells. To better visualise the cell membrane of such structures (black arrows), phase-contrast microscopy was utilised. An overlay of GP-1, F-actin and phase-contrast is provided within merged panels. B) TNT-like connections between 50 cell pairs were counted in both LCMV and mock infected cultures, with no statistical significance between resulting numbers.

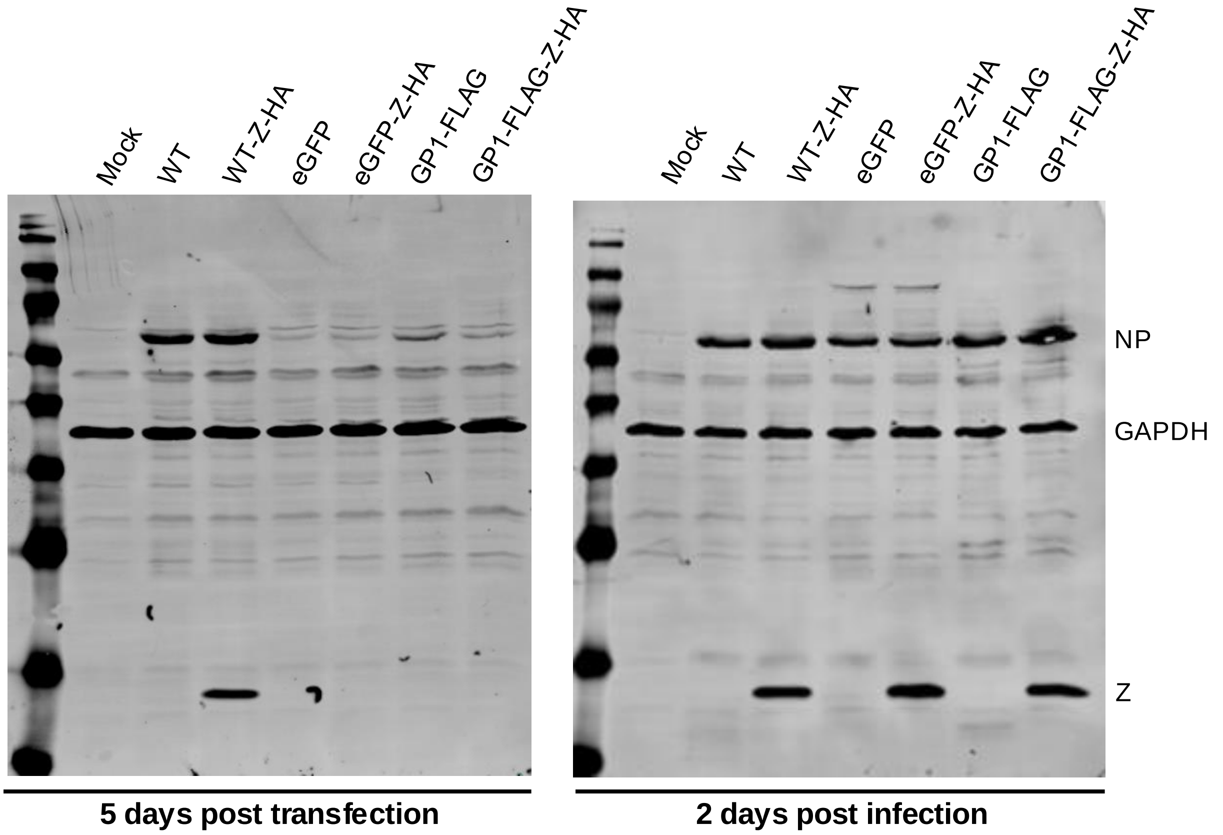

Figure S3. Uncropped Western blots of those shown in Figure 4C.

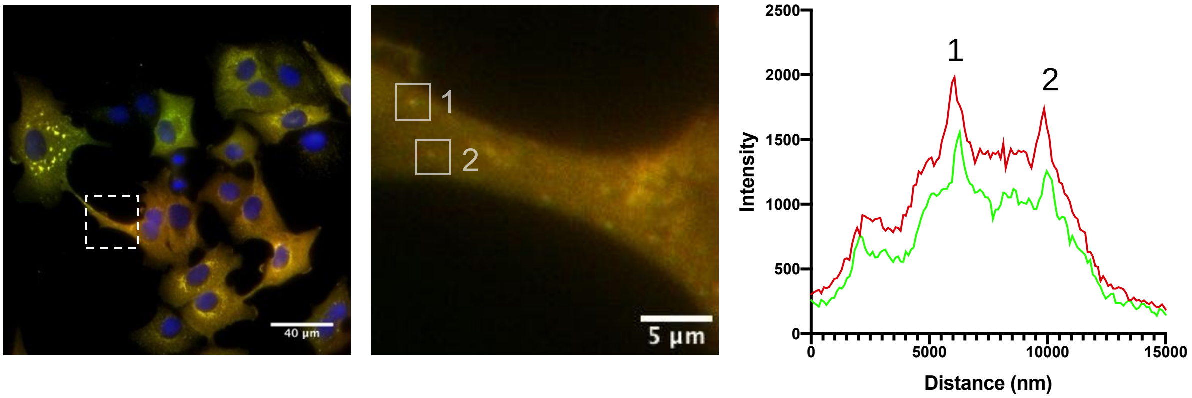

Figure S4. Representative TNT-like structures visualized within rLCMV-infected A549 cells, revealing co-localisation of LCMV NP (red) and S vRNA (green) as detected by FISH analysis. Line scan analysis was performed within the zoomed images represented by the dashed boxes, with the end points marked with the beginning (1) and end (2) of the scan line that correspond to the numbered peaks on the scan plot. Colours of the scan lines correspond to those in the cell images.

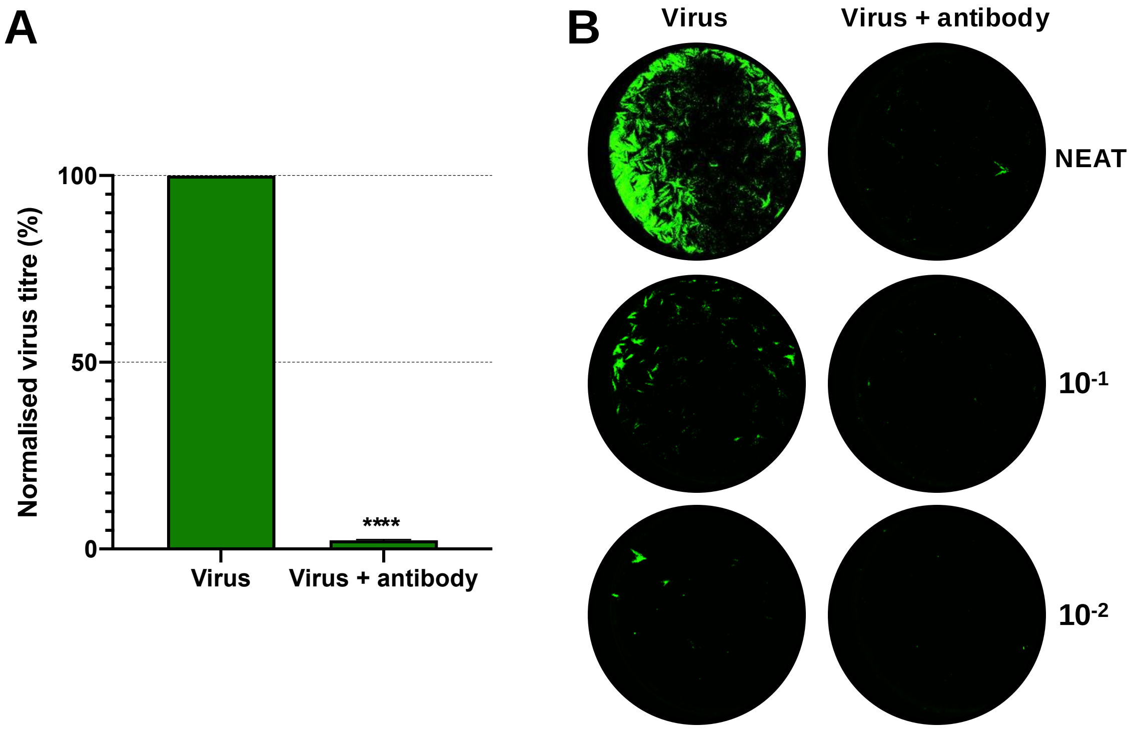

Figure S5. Analysis of neutralization ability of antibody M28 on extracellular viruses. (A) To examine the neutralising antibody effectiveness in neutralising virus released within Figure 7 experiment, viral supernatants at 18 hpi for virus only were collected. Subsequently, 5 µg/mL antibody was incubated with samples for 1 h and focus forming assays performed. The normalised viral titre is represented for virus with and without antibody, and the average of three independent experimental repeats is shown (n=3), with error bars showing standard deviation. (B) Representative images for virus and virus with antibody for the neat, 10^{-1} and 10^{-2} dilutions.
